# Impact of the 2022 American Academy of Pediatrics Hyperbilirubinemia Guideline on Phototherapy Utilization in a Resource-Limited Setting: A Single-Center Pre-Post Study

**DOI:** 10.7759/cureus.97846

**Published:** 2025-11-26

**Authors:** Vikram Sakaleshpur Kumar, Prajwal B Gadgeesh, Pratheeksha Naik, Gifty Mathew

**Affiliations:** 1 Pediatrics and Child Health, Subbaiah Institute of Medical Sciences, Shivamogga, IND; 2 Pediatric Medicine, Sarji Maternal and Child Hospital, Shivamogga, IND; 3 Pediatrics and Neonatology, Subbaiah Institute of Medical Sciences, Shivamogga, IND; 4 Pediatrics and Child Health, Sarji Maternal and Child Hospital, Shivamogga, IND; 5 Pediatrics and Neonatology, Sarji Maternal and Child Hospital, Shivamogga, IND

**Keywords:** aap 2022 guideline, hyperbilirubinemia, india, neonatal jaundice, newborn care, phototherapy, resource-limited setting

## Abstract

Background

The 2022 American Academy of Pediatrics (AAP) clinical practice guideline for neonatal hyperbilirubinemia raised phototherapy and exchange transfusion thresholds to minimize overtreatment. The real-world effect of these changes in resource-limited Indian settings remains unclear. This study aimed to evaluate the potential reduction in phototherapy use and validate the safety of the 2022 guideline.

Methodology

This single-center, quasi-experimental, pre-post study was conducted at Subbaiah Institute of Medical Sciences, Shivamogga, India. In part 1 (retrospective simulation), data from 100 consecutive term neonates (≥38 weeks' gestation) without neurotoxicity risk factors who received phototherapy under the 2004 AAP guideline were re-analyzed using the 2022 thresholds. In part 2 (prospective validation), another cohort of 100 consecutive term neonates without risk factors was prospectively managed using the 2022 guideline. Primary outcomes were phototherapy rate and readmission for jaundice within 14 days.

Results

Of 100 infants treated per the 2004 guideline, 54 (54%) had bilirubin levels below the 2022 phototherapy threshold and would not have required treatment. In the prospective 2022 cohort, only nine (9%) required phototherapy. No infant (0%) required readmission for jaundice.

Conclusions

Implementation of the 2022 AAP guideline reduced phototherapy use by over 50% compared to the 2004 criteria, without compromising safety. Adoption of the updated guideline in resource-limited settings can prevent unnecessary treatment, conserve resources, and support family-centered neonatal care.

## Introduction

Neonatal hyperbilirubinemia is among the most common conditions in the early newborn period. Concerns regarding bilirubin-induced neurologic dysfunction and kernicterus shaped the conservative treatment thresholds outlined in the 2004 American Academy of Pediatrics (AAP) guideline [1-4]. Visual assessment alone has since been shown to correlate poorly with serum bilirubin, especially across diverse skin tones, prompting greater reliance on objective measurement and predischarge risk assessment [5-8].

Over the past two decades, evidence from high-income settings has clarified that bilirubin neurotoxicity in healthy term infants occurs at higher bilirubin levels than previously assumed and that many infants treated under earlier guidelines may not have required phototherapy [9-16]. This led to the 2022 AAP update, which raised treatment thresholds and emphasized structured follow-up pathways [16-21]. Early evaluations have shown substantial reductions in phototherapy use without compromising safety.

However, the translation of this evidence to low- and middle-income countries (LMICs) remains uncertain. Severe jaundice continues to contribute disproportionately to neonatal morbidity and mortality in these settings, where gaps in timely bilirubin measurement, follow-up, and escalation of care persist [22-26]. Whether the higher 2022 thresholds can safely reduce overtreatment without increasing the risk of missed neurotoxicity in resource-limited contexts is still unknown.

To address this evidence gap, we assessed the potential reduction in phototherapy use when applying the 2022 AAP guideline in a resource-constrained setting and evaluated short-term safety following its prospective implementation.

## Materials and methods

Study design and setting

This was a single-center, quasi-experimental, pre-post quality improvement (QI) study conducted in the neonatal unit of the Department of Pediatrics, Subbaiah Institute of Medical Sciences, Shivamogga, India. The project consisted of two phases: (1) a retrospective simulation applying the 2022 guideline to infants treated under the 2004 criteria and (2) a prospective implementation phase evaluating real-world outcomes after adoption of the 2022 guideline.

Feasibility and context

Given the resource-limited setting, the study was intentionally designed to require minimal additional infrastructure. Earlier evidence has demonstrated the feasibility of non-hospital phototherapy in appropriately selected infants and highlighted the importance of efficient resource utilization in neonatal care [13]. Subsequent systematic evaluations have reinforced that structured, reproducible implementation methods are essential for safe adoption of clinical practice changes [14,15].

Quality improvement framework

The project was guided by the Plan-Do-Study-Act (PDSA) model, a well-established iterative framework for healthcare quality improvement [12].

Plan

High phototherapy rates and potential overtreatment were identified as targets for improvement.

Do

Staff were trained on the 2022 AAP hyperbilirubinemia guideline, visual decision aids were introduced, and the new thresholds were incorporated into routine practice.

Study

Phototherapy utilization and readmission rates were monitored prospectively.

Act

Based on the results, adoption of the 2022 guideline became the recommended departmental practice.

Foundational work on QI science emphasizes that iterative learning cycles and multidisciplinary engagement improve reliability and sustainability of clinical processes [15].

Ethical considerations

The Institutional Ethics Committee of Subbaiah Institute of Medical Sciences approved the study on October 10, 2025 (approval number: IEC-SUIMS/184/2025-26). Because this was a quality improvement project utilizing de-identified clinical data, the requirement for individual informed consent was waived.

Study population

Eligible participants were term infants (≥38 weeks of gestation) without neurotoxicity risk factors as defined in the 2022 AAP guideline. Infants were excluded if they had hemolysis, glucose-6-phosphate dehydrogenase (G6PD) deficiency, sepsis, lethargy, temperature instability, metabolic acidosis, serum albumin < 3 g/dL, direct hyperbilirubinemia, or major congenital anomalies.

In the Retrospective phase, 100 infants who received phototherapy under the 2004 guideline (January-December 2023) were included. In the prospective phase, 100 infants managed using the 2022 guideline (February-August 2024) were included.

Description of the intervention

The intervention consisted of formally replacing the 2004 AAP hyperbilirubinemia guideline with the updated 2022 guideline [24,26]. Staff received a structured one-hour orientation, and laminated threshold charts were displayed in clinical areas. To ensure consistency, operational differences between the 2004 and 2022 guidelines, particularly treatment thresholds, neurotoxicity risk factor definitions, and escalation of care criteria, were summarized for staff education. The key distinctions informing the intervention are shown in Table 1.

**Table 1:** Key differences between the 2004 and 2022 AAP hyperbilirubinemia guidelines AAP: American Academy of Pediatrics, TcB: transcutaneous bilirubin, TSB: total serum bilirubin, IV: intravenous

Feature	2004 AAP charts	2022 AAP charts	Rationale for the change
Phototherapy thresholds	Lower and more conservative thresholds	Narrowly higher thresholds for initiating phototherapy	Based on 18 years of new evidence showing that bilirubin neurotoxicity occurs at higher levels than previously thought, reducing unnecessary phototherapy and hospitalizations
Risk categories	Used three risk categories based on a combination of gestational age and risk factors	Separated gestational age from neurotoxicity risk factors for more specific guidance	Allows for more precise, individualized assessment of treatment needs
Risk factor assessment	Used risk factors to assess the risk of developing severe hyperbilirubinemia	Shifted focus to neurotoxicity risk factors; factors remain similar, but the guidelines clarify their use in lowering treatment thresholds	More directly addresses the risk of bilirubin-induced neurologic dysfunction
Race-based factors	Included "Black race" as a lower risk factor for significant jaundice based on older studies	Eliminated race as a risk factor	Newer data show Black infants have an increased risk of severe hyperbilirubinemia and that older studies were flawed; the change addresses health equity concerns
Screening policy	Less specific guidance on universal screening	Recommends universal bilirubin screening for all newborns (≥35 weeks) using TcB or TSB before discharge	Ensures early identification and reduces reliance on inaccurate visual assessment
Screening methods	More limited guidance on TcB	Emphasizes TcB as a reliable screening tool but confirms that TSB is the definitive measure for treatment decisions	Reduces the need for invasive blood draws while providing effective screening
Post-discharge follow-up	Based on a nomogram with risk zones	Uses the difference between the infant's bilirubin concentration and the phototherapy threshold to determine the timing of follow-up	Provides a more evidence-based approach to follow-up, based on a baby's individual risk
Escalation of care	Lacked a specific, defined concept for managing worsening cases	Formally introduced the concept of "escalation of care" for infants with rapidly rising bilirubin levels; this includes specific steps like IV hydration, intensive phototherapy, and transfer to a facility for exchange transfusion	Streamlines the process for treating severe hyperbilirubinemia to prevent kernicterus
Clinical outcomes	Higher rates of phototherapy use and hospitalization	Studies following the new guidelines show a significant reduction in phototherapy and hospital admissions without an increase in neurotoxicity	Supports the success of the updated, less aggressive thresholds

Outcome measures

Two primary metrics were evaluated: retrospective (pre-intervention), the proportion of infants whose peak total serum bilirubin (TSB) would not have met 2022 phototherapy criteria (potential overtreatment), and prospective (post-intervention), the proportion requiring phototherapy under the 2022 guideline and 14-day readmission rate for jaundice or phototherapy.

Secondary descriptors included the distribution of bilirubin values relative to 2022 thresholds.

Bilirubin assessment

Clinical assessment of jaundice was supplemented by total serum bilirubin measurements. Prior evidence has demonstrated limitations of visual jaundice assessment and emphasized the need for objective bilirubin evaluation, particularly in diverse populations [22,23]. Transcutaneous bilirubinometry was not used due to equipment constraints.

Statistical analysis

Data were analyzed using descriptive statistics (proportions and percentages). Inferential statistical tests were not applied because the two cohorts represented non-overlapping time periods, and the study objective was quality improvement rather than hypothesis testing.

## Results

Part 1: Retrospective simulation (pre-intervention)

A total of 100 term infants without neurotoxicity risk factors who received phototherapy under the 2004 AAP guideline were included in the retrospective phase. When the 2022 AAP thresholds were retrospectively applied to these infants, 54 out of 100 (54%) had peak total serum bilirubin (TSB) levels below the updated phototherapy threshold.

This indicates that more than half of the infants treated with phototherapy under the older guideline would not have met criteria for treatment under the 2022 guideline, suggesting a substantial potential for overtreatment. Similar findings have been reported in retrospective simulations from Italy, Turkey, and other regions, where 40%-80% of infants who received phototherapy per older thresholds would not have required treatment under updated criteria.

**Figure 1:**
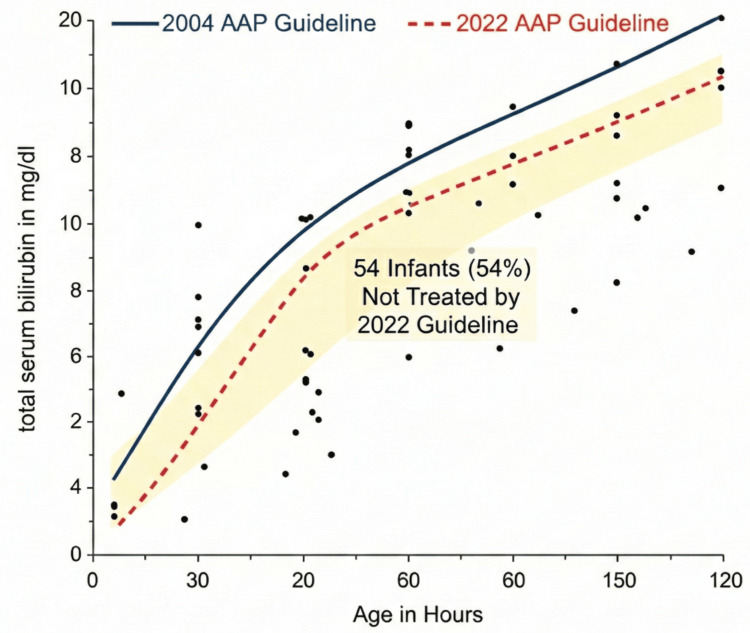
Retrospective simulation of the 2022 AAP guideline on 100 infants treated per the 2004 guideline The plot shows 100 infants (black dots) treated per the 2004 AAP guideline (solid blue line). The 2022 AAP guideline (dashed red line) is also shown. The 54 infants (54%) who fall into the "gap" between the two lines (shaded yellow) represent the cohort that would not have received phototherapy under the new guidelines. AAP: American Academy of Pediatrics

A graphical illustration of the retrospective simulation is shown in Figure 1, demonstrating the distribution of bilirubin values in comparison with the 2004 and 2022 guideline thresholds.

Part 2: Prospective validation (post-intervention)

During the prospective phase, 100 consecutive term infants were evaluated and managed using the 2022 AAP hyperbilirubinemia guideline.

Phototherapy Utilization

A total of nine (9%) infants required phototherapy, while 91 (91%) infants were managed conservatively without treatment. These findings represent a substantial reduction in phototherapy use compared to the retrospective cohort.

The distribution of outcomes is summarized in Table 2, which highlights the markedly lower phototherapy initiation rate under the updated guideline.

**Table 2:** Clinical outcomes of prospective cohort (N=100) managed using the 2022 AAP guideline The primary safety outcome was met. Of the 100 infants in the prospective cohort, including the 91 who were not treated, 0 (0%) required readmission to the hospital for phototherapy or elevated jaundice. The outcomes of this prospective cohort are summarized in the table.

Outcome	Number of infants (N=100)	Percentage
Phototherapy initiated	9	9%
Phototherapy not required	91	91%
Hospital readmission for jaundice	0	0%

Safety Outcomes

No infants (0%) required hospital readmission for jaundice or phototherapy within 14 days of discharge. The absence of readmissions indicates that the higher phototherapy thresholds specified in the 2022 guideline did not compromise short-term clinical safety in this setting.

Summary of Findings

As shown in Table 2, the prospective application of the updated guideline resulted in low phototherapy utilization (9%), a high proportion of infants safely managed without phototherapy (91%), and zero readmissions (0%) within 14 days.

These outcomes demonstrate both the feasibility and safety of adopting the 2022 guideline in a resource-limited setting.

## Discussion

This quality improvement study demonstrates that adoption of the 2022 American Academy of Pediatrics (AAP) hyperbilirubinemia guideline can substantially reduce phototherapy utilization in a resource-limited setting without compromising short-term safety. Retrospective application of the updated thresholds revealed that more than half of the infants treated under the 2004 guideline would not have required phototherapy, echoing early foundational concerns that conservative treatment thresholds may contribute to unnecessary intervention [1]. Understanding bilirubin neurotoxicity and the spectrum of bilirubin-induced neurologic dysfunction has evolved considerably since the mid-2000s [2-4], with subsequent work refining the balance between preventing undertreatment and avoiding overtreatment.

Limitations of visual jaundice assessment, well documented across multiple studies, further highlight the importance of relying on objective bilirubin measurement rather than clinical impression alone [5-8]. As early as 2009, structured investigations demonstrated that visual estimation performs poorly across gestational ages and skin tones [5], reinforcing the value of standardized thresholds and risk-based surveillance. Reliable bilirubin assessment became even more central as large predischarge screening studies in the following decade showed that many infants were being treated at bilirubin levels below what later evidence suggested was necessary [10,11].

By the mid-2010s, systematic reviews and feasibility studies, ranging from home phototherapy evaluations to structured quality improvement research, highlighted persistent variation in care and opportunities to reduce unnecessary hospitalization while maintaining safety [12-15]. A growing body of global epidemiologic evidence also underscored the disproportionate burden of severe neonatal jaundice in low- and middle-income countries (LMICs), largely driven by delayed detection and inconsistent follow-up [16-19]. These contextual realities reinforced the need for a clearer, evidence-based framework that could be applied across diverse resource settings.

The 2022 AAP guideline revision incorporated nearly two decades of emerging evidence and recalibrated treatment thresholds accordingly, emphasizing clearer neurotoxicity risk definitions, higher phototherapy thresholds for healthy term infants, and strengthened follow-up pathways [24,26]. Subsequent analyses from 2023 onward have reinforced the rationale for these changes, including updated reviews of clinical management practices and global burden estimates [25-28].

Comparison with published studies

Implementation studies following the release of the 2022 guideline have consistently reported large reductions in phototherapy utilization. Early evaluations in high-resource settings demonstrated decreases of 40%-50% with no increase in severe hyperbilirubinemia or exchange transfusion [29]. Similar reductions have been reported in LMIC and middle-resource contexts examining home phototherapy models and optimized follow-up strategies [30-33]. A comparative summary of major post-guideline studies from the United States, Italy, and Türkiye is presented in Table 3, illustrating a consistent international pattern: lower phototherapy rates without measurable compromise in safety outcomes.

**Table 3:** Comparative evidence from post-2022 implementation studies IVIG: intravenous immunoglobulin

Study	Country	Sample size	Design	Change in phototherapy	Safety outcome
Sarathy et al. (2024) [29]	USA	>22,000	Multicenter pre-post	↓ 47% (3.9% → 2.1%); bilirubin draws ↓ 23%	No rise in readmissions or kernicterus
Jameel et al. (2025) [30]	USA	Nationwide PHIS	Interrupted time series	Hospitalizations ↓ from 5,051 to 3,778	No change in IVIG/exchange/kernicterus
Dani et al. (2025) [31]	Italy	876	Retrospective simulation	82% below 2022 thresholds → 42%-68% avoidable admissions	No increase in rebound cases
Demirtaş et al. (2025) [33]	Türkiye	582	Single-center pre-post	Hospitalization ↓ (23% → 14%)	No neurotoxicity events reported

Our findings fall squarely within this global range. The retrospective cohort suggested a 54% theoretical reduction in phototherapy, while the prospective implementation phase demonstrated an actual treatment rate of 9%, lower than many published series. Importantly, no infants in the prospective cohort required readmission for jaundice, supporting the short-term safety of adopting the updated thresholds even in a resource-limited clinical environment.

Implications for low- and middle-income countries

In LMIC contexts, where hospital resources, nursing time, and laboratory capacity are often constrained, minimizing clinically unnecessary phototherapy has substantial operational value. Earlier research has shown that a significant share of admissions for jaundice may be preventable with appropriate threshold adjustments and structured follow-up [16-18]. Our results support this position, demonstrating that safe reduction in treatment burden is achievable with careful planning, consistent training, and clear decision aids [19-21].

Safety considerations

Population-level surveillance following adoption of the 2022 guideline has not shown increases in severe hyperbilirubinemia outcomes, including exchange transfusion or kernicterus [22-27]. Reported kernicterus incidence remains extremely low and typically arises in infants with hemolysis, prematurity, or delayed care [2,7,10]. Ensuring universal bilirubin screening and reliable follow-up remains crucial in LMIC settings.

Strengths and limitations

The strengths of this study include its pragmatic, real-world design, structured PDSA methodology, and demonstration of feasibility without additional equipment or staffing. Limitations include the single-center scope, modest sample size, and lack of long-term neurodevelopmental follow-up. Nonetheless, the consistency between our findings and the broader international evidence enhances the validity and generalizability of the results.

Key lessons

The 2022 AAP guideline provides a rational, evidence-based framework for reducing overtreatment. When implemented through structured QI processes, it can be safely adapted even in resource-constrained settings. Ongoing caregiver education and standardized follow-up remain essential.

Future directions

Future multicenter studies in LMICs are needed to quantify cost savings, assess long-term outcomes, and evaluate scalable models for integrating transcutaneous bilirubin (TcB) screening and digital decision support tools into routine newborn care. Such work will be essential to ensuring equitable implementation of evidence-based hyperbilirubinemia management across diverse clinical environments.

## Conclusions

Implementation of the 2022 American Academy of Pediatrics hyperbilirubinemia guideline in a resource-limited setting resulted in a substantial reduction in phototherapy use without compromising short-term safety. Both the retrospective simulation and the prospective implementation phases demonstrated that many infants previously treated under older thresholds did not require intervention when evaluated under the updated criteria. These findings support the safety, feasibility, and operational value of adopting the 2022 guideline in similar low- and middle-income settings, where reducing unnecessary phototherapy can conserve scarce clinical resources and minimize family disruption. Sustained improvement will depend on structured follow-up, continued staff education, and system-level integration of reliable bilirubin assessment. Broader multicenter evaluations, including long-term outcomes, are now warranted to inform national implementation strategies and strengthen evidence-based neonatal care across diverse healthcare environments.
